# Non-targeted Metabolomics Reveals Metabolic Characteristics of Porcine Atretic Follicles

**DOI:** 10.3389/fvets.2021.679947

**Published:** 2021-07-26

**Authors:** Jiayuan Mo, Le Sun, Juanru Cheng, Yujie Lu, Yaochang Wei, Guangsheng Qin, Jing Liang, Ganqiu Lan

**Affiliations:** ^1^College of Animal Science and Technology, Guangxi University, Nanning, China; ^2^Key Laboratory of Buffalo Genetics, Guangxi Buffalo Research Institute, Chinese Academy of Agricultural Science, Nanning, China

**Keywords:** Bama Xiang pig, follicular atresia, non-targeted metabolomics, LC-MS, follicular fluid

## Abstract

Follicular atresia is one of the main factors limiting the reproductive power of domestic animals. At present, the molecular mechanisms involved in porcine follicular atresia at the metabolic level remain unclear. In this study, we divided the follicles of Bama Xiang pigs into healthy follicles (HFs) and atretic follicles (AFs) based on the follicle morphology. The expression of genes related to atresia in granulosa cells (GCs) and the concentration of hormones in the follicular fluid (FF) from HFs and AFs were detected. We then used liquid chromatography–mass spectrometry-based non-targeted metabolomic approach to analyze the metabolites in the FF from HFs and AFs. The results showed that the content of estradiol was significantly lower in AFs than in HFs, whereas that of progesterone was significantly higher in AFs than that in HFs. The expression of *BCL2, VEGFA*, and *CYP19A1* was significantly higher in HFs than in AFs. In contrast, the expression of *BAX* and *CASPASE3* was significantly lower in HFs. A total of 18 differential metabolites (DMs) were identified, including phospholipids, bioactive substances, and amino acids. The DMs were involved in 12 metabolic pathways, including arginine biosynthesis and primary bile acid biosynthesis. The levels of eight DMs were higher in the HF group than those in the AF group (*p* < 0.01), and those of 10 DMs were higher in the AF group than those in the HF group (*p* < 0.01). These findings indicate that the metabolic characteristics of porcine AFs are lower levels of lipids such as phospholipids and higher levels of amino acids and bile acids than those in HFs. Disorders of amino acid metabolism and cholic acid metabolism may contribute to porcine follicular atresia.

## Introduction

Reproductive ability, which is a base index, typically refers to productivity in the porcine industry. In mammals, the ovary produces generative cells. The number of follicles is an index that determines the reproductive ability of female animals, especially polyembryonic animals. However, when more than 90% of the follicles degenerate before ovulation, the condition is termed follicular atresia (1). It is well-known that follicular cells include not only oocytes but also follicular fluid (FF) and granulosa cells (GCs). Many factors affect follicular atresia; however, GC apoptosis is the main molecular mechanism (2, 3). Additionally, the physiological activities of the hypothalamus–pituitary–gonadal axis occupy an influential position in follicular atresia (4). The molecules in FF, such as hormones [including melatonin (5) and growth hormone (6)] and cytokines [transforming growth factor beta (TGF-β) superfamily (7), insulin-like growth factor (IGF) (8), interleukin-6 (IL6) (9), and epidermal growth factor (EGF) (10)], also affect follicular atresia. Because of the transfer of plasma and the secretion of follicle cells, the FF component is highly complex. The concentration of FF components may be inconsistent in diverse follicular cell types, especially between atretic follicles (AFs) and healthy follicles (HFs). Nishimoto et al. (11) studied the difference between HFs and AFs in cattle and proposed that glucose and lactic acid may be markers of follicular atresia in FF. However, the consistency of these indices in Bama Xiang pigs is unknown.

Fortunately, sequencing technology has quickly developed with the development of biotechnology and computer technology. After RNA sequencing (RNA-seq) explained the difference at the transcription level, proteomics explained the difference at the translation level. Metabolomics is an emerging omics technology that explains differences at the metabolic level. We can perform qualitative and quantitative analyses of all small molecule metabolites (<1 kDa) in specific biological samples (such as blood, urine, and FF) by metabolomics. This technology is suitable for the study of physiological mechanisms and the discovery of biomarkers (12). At present, metabolomics based on FF samples have shown great potential in reproductive medicine. It is used to identify biomarkers that assess oocyte quality to predict the outcome of assisted reproduction (13). Additionally, metabolomic analysis of FF screened out biomarkers of multiple reproductive diseases such as polycystic ovary syndrome (14), endometriosis (10), and infertility (15) and characterized their global metabolic profile to provide a reference for the clinical diagnosis of these diseases and an in-depth study of their pathogenesis. This study indicated that this method can be used to identify biomarkers in animal husbandry and veterinary medicine.

Bama Xiang pigs are a Chinese indigenous pig and are famous for their small size, excellent meat quality, and early maturation (16). Bama Xiang boars produce sperm in the seminiferous tubules at the age of 29 days, and the sex maturation time is 76 days. For Bama Xiang sows, the sex maturation time is 110 days (17). However, the litter size of Bama Xiang pigs is lower than that of many Chinese indigenous breeds, such as Meishan pigs, Bamei pigs, and Erhualian pigs. Therefore, using metabolomics to identify biomarkers between HFs and AFs will be of great benefit in improving the understanding of Bama Xiang pig reproduction traits. Meanwhile, because of the parallels with humans in anatomy and physiology, Bama Xiang pigs could be used in human medical research (18–21). Our study used a liquid chromatography–mass spectrometry (LC–MS)-based non-targeted metabolomic approach for the first time to analyze the FF of HFs and AFs in Bama Xiang pigs. The results will further increase the understanding of the mechanism of follicular atresia in Bama Xiang pigs and provide a reference for experimental animals to benefit human reproduction studies.

## Materials and Methods

### Follicle Classification

A total of 10 ovaries were collected from the right side of 6-month-old Bama Xiang pigs from the Bama Xiang Pig Agriculture and Animal Husbandry Co., Ltd. (Guangxi, China). After collection, the ovaries were rinsed with physiological saline, placed in a heat preservation pot containing physiological saline at 37°C with high-pressure processing, and transported to the laboratory within 2 h. After rinsing with 75% ethanol, the ovaries were washed with physiological saline at 37°C. Ophthalmic scissors were used to cut open and separate a single follicle (3 mm < *n* < 5 mm) from the ovarian tissue. The single follicle was repeatedly rinsed with 0.1 M phosphate buffer (pH 7.25). As previously mentioned (22), we divided the follicles into HF and AF groups based on follicle morphology. The morphology of the follicle mainly included the following two aspects: the appearance of the follicle (such as color, capillary, and transparency) (23) and state of cumulus–oocyte complex (COC) and GCs.

### Collection of Granulosa Cells and Follicular Fluid

After morphological classification, we removed the oocytes, and the remaining liquids (including FF and GCs) were mixed. The FF and GCs were separated by centrifugation (4°C, 1,200 rpm, 5 min). After washing 2–3 times with precooled phosphate buffered saline, the collected GCs were mixed with 1 ml TRIzol and stored at −80°C. The FF was centrifuged again (4°C, 8,000 rpm, 5 min) to collect the supernatant. Then, 30 μl of the supernatant was used to detect the concentration of estradiol (E2) and progesterone (PROG), and the rest was stored at −80°C for LC–MS analysis. In the same individual, we collected only one HF and one AF sample. A total of 10 samples each were collected from the HF group and AF group.

### Measurements of Estradiol and Progesterone

The hormone levels related to follicular atresia, such as E2 (24) and PROG (25) in FF, were measured using ELISA kits (Shanghai Enzyme Biotechnology Co., Ltd., China, ml002366, ml002422) to confirm the follicle classification. A total of 20 FF samples were analyzed using a microplate reader (Infinite M200 PRO, TECAN) according to the manufacturer's instructions. All assays were validated in our laboratory by showing parallels between serial sample dilutions and the provided assay standard curve (range: E2, 2.5–80 pg/ml; PROG, 0.625–20 ng/ml). Sensitivity of the E2 and PROG assays was 0.1 pg/ml and 0.1 ng/ml, respectively. The intra-assay coefficient of variation was <15% for each.

### cDNA Synthesis and Quantitative RT-PCR for Related Genes

The TRIzol Reagent (Ambion, Life Technologies, New York, USA), chloroform, isopropanol, and ethanol were used to extract the total RNA. RNase-free water was used to dissolve total RNA from GCs. Oligo (dT)_23_VN (10 μM), 4× gDNA wiper Mix, and RNase-free ddH_2_O at 42°C were used for 2 min to remove the DNA from the GCs. We used the 5× HiScript II Select qRT SuperMix II at 50°C for 15 min to convert the RNA into cDNA (Vazyme, R223-01, China) (26). Prior to quantitative RT-PCR (qRT-PCR) amplification, the sequences of primers for *BAX, BCL2, CASPASE3, CYP19A1, VEGFA*, and *GAPDH* (Table 1) were designed using the Oligo 7.0 software. qRT-PCR was performed on an Applied Biosystems 7500 Real-Time PCR System (ABI7500; Applied Biosystems, Carlsbad, CA, USA) using ChamQ Universal SYBR qPCR Master Mix (Vazyme, Q711-02/03, China) following the manufacturer's instructions. The relative expression of each gene was calculated using the 2^−ΔΔCt^ method with *GAPDH* as the reference gene.

**Table 1:** Quantitative real-time PCR primers of genes.

**Name**	**Primer sequence (5′-3′)**	**Length (bp)**	**Reference/accession numbers**
*BAX*-F	GCCGAAATGTTTGCTGAC	154	AJ606301
*BAX*-R	GCCGATCTCGAAGGAAGT		
*BCL2*-F	GATGCCTTTGTGGAGCTGTATG	145	AB271960
*BCL2*-R	CCCGTGGACTTCACTTATGG		
*CASPASE3*-F	TGGATGCTGCAAATCTCA	327	NM_214131
*CASPASE3*-R	TCCCACTGTCCGTCTCAA		
*CYP19A1*-F	GCTGCTCATTGGCTTAC	187	NM_214431
*CYP19A1*-R	TCCACCTATCCAGACCC		
*VEGFA*-F	CCTTGCTGCTCTACCTCC	239	NM_214084
*VEGFA*-R	CTCCAGACCTTCGTCGTT		
*GAPDH*-F	GGACTCATGACCACGGTCCAT	220	AF017079
*GAPDH*-R	TCAGATCCACAACCGACACGT		

### Follicular Fluid Sample Preparation

Prior to FF metabolite analysis, a 50-μl FF sample with 125 μl of methanol and 125 μl of acetonitrile was added to 1.5-ml Eppendorf (EP) tubes, vortexed for 30 s to precipitate protein, underwent a low-temperature ultrasound for 15 min, and then was centrifuged (13,000 rpm, 20 min, 4°C). The supernatant fractions were collected and evaporated using a vacuum concentrator. The resulting dry residues were redissolved in 50 μl of acetonitrile:water (1:1) and centrifuged again (13,000 rpm, 20 min, 4°C). Finally, the liquid supernatant was transferred to sampler vials for analysis using a ultraperformance LC–MS (UPLC-MS) system (27).

### Ultraperformance Liquid Chromatography–Mass Spectrometry Instrument and Parameters

A Dionex liquid chromatograph instrument (UltiMate3000, Thermo Fisher Scientific, USA) was utilized. It contained a liquid phase pump (HPG-3400 SD, Thermo Fisher Scientific, USA), column temperature box (TCC-3000 SD, Thermo Fisher Scientific, USA), and an autosampler (WPS-3000SL, Thermo Fisher Scientific, USA). An ACQUITY UPLCBEHC18 column (50 × 2.1 mm, 1.7 μm) was also used. The ion source was a quadrupole electrostatic field orbit trap high-resolution mass spectrometer Q-Exactive (Thermo Fisher Scientific, USA) heated electrospray (Thermo Fisher Scientific, USA). The scanning mode was full MS and full MS/dd-MS2 (Table 2) (27).

**Table 2:** Instrument operation program of UPLC-MS/MS analysis.

**Item**	**Parameter**	**Item**	**Parameter**
Column temperature	30°C	Mass range	80–1,200 m/z
Autosampler temperature	4°C	Sheath gas flow rate	30 psi
Sample injection volume	2 μl	Auxiliary gas flow rate	10 psi
Mobile phase A	Water plus 0.1% formic acid	Transmission Capillary temperature	320°C
Mobile phase B	Methanol	Primary scan resolutions	70,000
Spray voltage	3.0 kV	Secondary scan resolutions	17,500

### Data Analysis

The original data were processed using the Compound Discover 3.1 software for peak alignment of metabolites, to correct the retention time of metabolites, and to extract the peak area of metabolites. To identify the metabolite structures, accurate mass matching (<25 ppm) and matching with secondary spectra were used in the experiment. The peak areas of all metabolites were imported into the SIMCA-P program (version 14.1, Umetrics) for multivariate analysis, consisting of unsupervised principal component analysis (PCA) and supervised orthogonal partial least squares discriminant analysis (OPLS-DA). PCA was used to determine intragroup aggregation and inter-group separation tendencies, whereas OPLS-DA was performed to further determine inter-group differences. The OPLS-DA models were validated based on the interpretation of variation in Y (R2Y) and forecast ability based on the model (Q2) in cross-validation and permutation tests by applying 200 iterations. Univariate analyses were performed, including independent-samples *t*-test and fold change analysis. Significant differential metabolites (DMs) were screened using variable importance in projection (VIP) scores (VIP > 1) obtained from the OPLS-DA model and *p*-values (*p* < 0.05) using the *t*-test. In addition, the metabolic pathway analysis network tools MetPA (http://metpa.metabolomics.ca), MetaboAnalyst (https://www.metaboanalyst.ca/MetaboAnalyst/home.xhtml), and the Encyclopedia of Genes and Genomes in the Kyoto Protocol (KEGG, http://www.genome.jp/kegg/) were used to identify potentially disordered metabolic pathways. The differences between groups for hormone concentration and the qRT-PCR results were analyzed using independent-samples *t*-test analysis in SAS 9.2, and the results are expressed as the mean ± standard error of the mean. Unless otherwise stated, differences were considered statistically significant at *p* < 0.05.

## Results

### Appearance of Follicles

**Figure 1:**
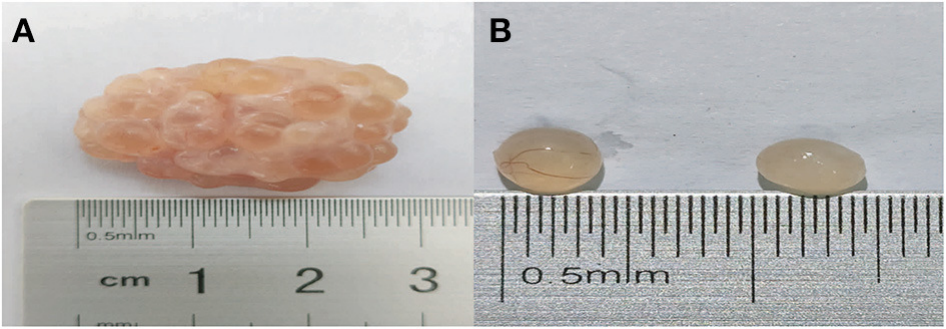
The morphologies of the ovary and follicles of Bama Xiang pig. **(A)** The right ovary of the Bama Xiang pig. **(B)** The healthy follicle (left side) and atretic follicle (right side) in the same ovary of Bama Xiang pig.

We identified one HF and one AF from the same ovary. The COCs, GCs, and FF were noticeably different between HFs and AFs. The cumulus, ooplasm, zona pellucida, GCs, and FF were damaged or deteriorated in AFs (Table 3). In follicles of the same size, more blood vessels were observed in the HFs than in the AFs (Figure 1).

**Table 3:** Classification of follicles based on the state of COCs and granulosa cells.

**Classification**	**COCs**	**GCs**	**FF**
HF	Compact cumulus and translucent ooplasm	Large quantity and compact	Clear
AF	Strongly extended or absent cumulus and dark ooplasm, and damaged zona pellucida	Few quantity and Quicksand shaped	Turbid

### Determination of Estradiol and Progesterone in Follicular Fluid

**Figure 2:**
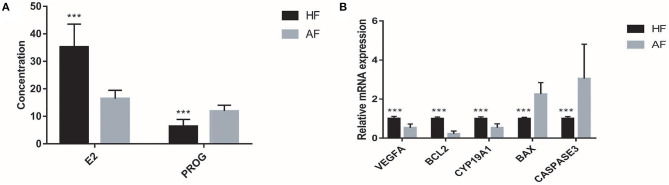
The hormone contents and gene expressions in the healthy follicle (HF) and atretic follicle (AF) samples. **(A)** The contents of estradiol (E2) and progesterone (PROG) in the follicular fluid (FF) from HF and AF, respectively. **(B)** The relative mRNA expressions of *VEGFA, BCL2, CYP19A1, BAX*, and *CASPASE3* genes in the HF and AF samples, respectively. *** highly significant difference between the two groups (*p* < 0.01).

Each sample in the HF and AF groups was analyzed three times. A total of 60 records of E2 and PROG were used to carry out the independent-samples *t*-test analysis in SAS 9.2. The concentrations of E2 in the HF and AF were 35.20 ± 8.30 and 16.43 ± 3.06 pg/ml, respectively. The concentrations of PROG in the HF and AF were 6.41 ± 2.45 and 11.89 ± 2.14 ng/ml, respectively. Compared with the HF, in the AF, the content of E2 in the FF was significantly reduced (*p* < 0.01) and that of PROG was significantly increased (*p* < 0.01) (Figure 2A).

### Expressions of Genes Related to Atresia in Granulosa Cells

Similar to the content of E2 and PROG, the expression of genes related to atresia in GCs from the HF and AF was assessed three times in each sample. A total of 60 records of each gene from the 20 samples, including *BCL2, VEGFA, CYP19A1, BAX*, and *CASPASE3*, were used to carry out the independent-samples *t*-test analysis using SAS 9.2 software. While we used the gene expression of HF as 1, the expression levels of *BCL2, VEGFA, CYP19A1, BAX*, and *CASPASE3* in AF were 0.22 ± 0.14, 0.53 ± 0.19, 0.53 ± 0.20, 2.26 ± 0.59, and 3.05 ± 1.76, respectively. The expression of *BCL2, VEGFA*, and *CYP19A1* in HF was significantly higher than that in AF (*p* < 0.01). The expression of *BAX* and *CASPASE3* in HF was significantly lower than that in AF (*p* < 0.01) (Figure 2B).

### Metabolite Profiles of Follicular Fluid

**Figure 3:**
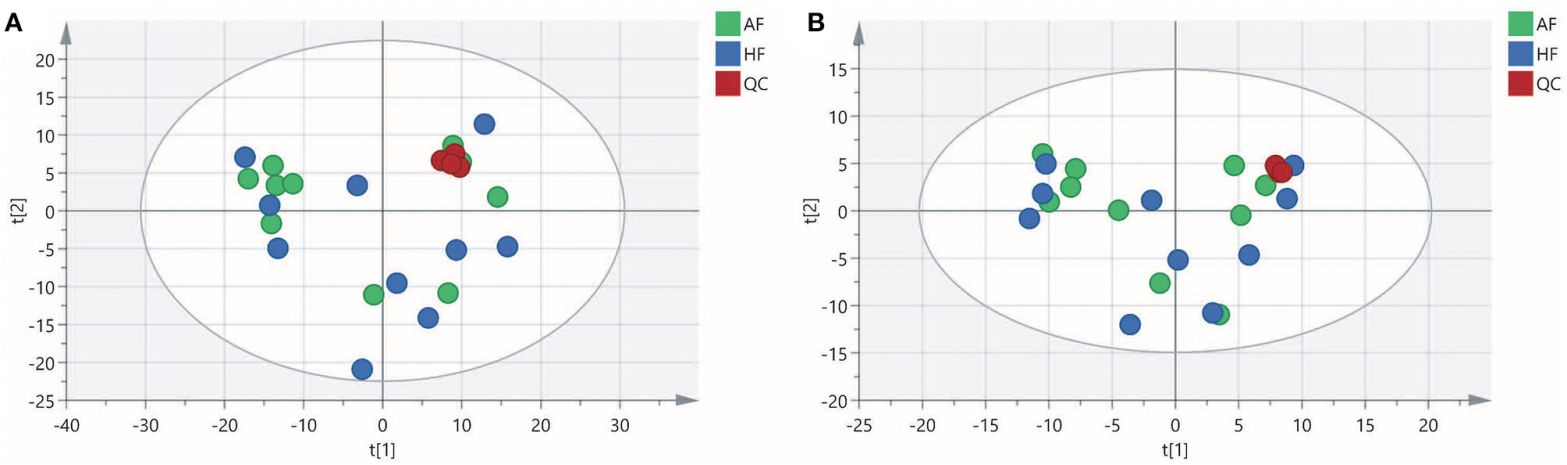
Principal component analysis (PCA) score plot of the healthy follicle (HF) and atretic follicle (AF) groups. **(A)** PCA score plot for the two groups analyzed in the positive ion mode. **(B)** PCA score plot for the two groups analyzed in the negative ion mode. t[1] was the first principal component; t[2] was the second principal component.

**Figure 4:**
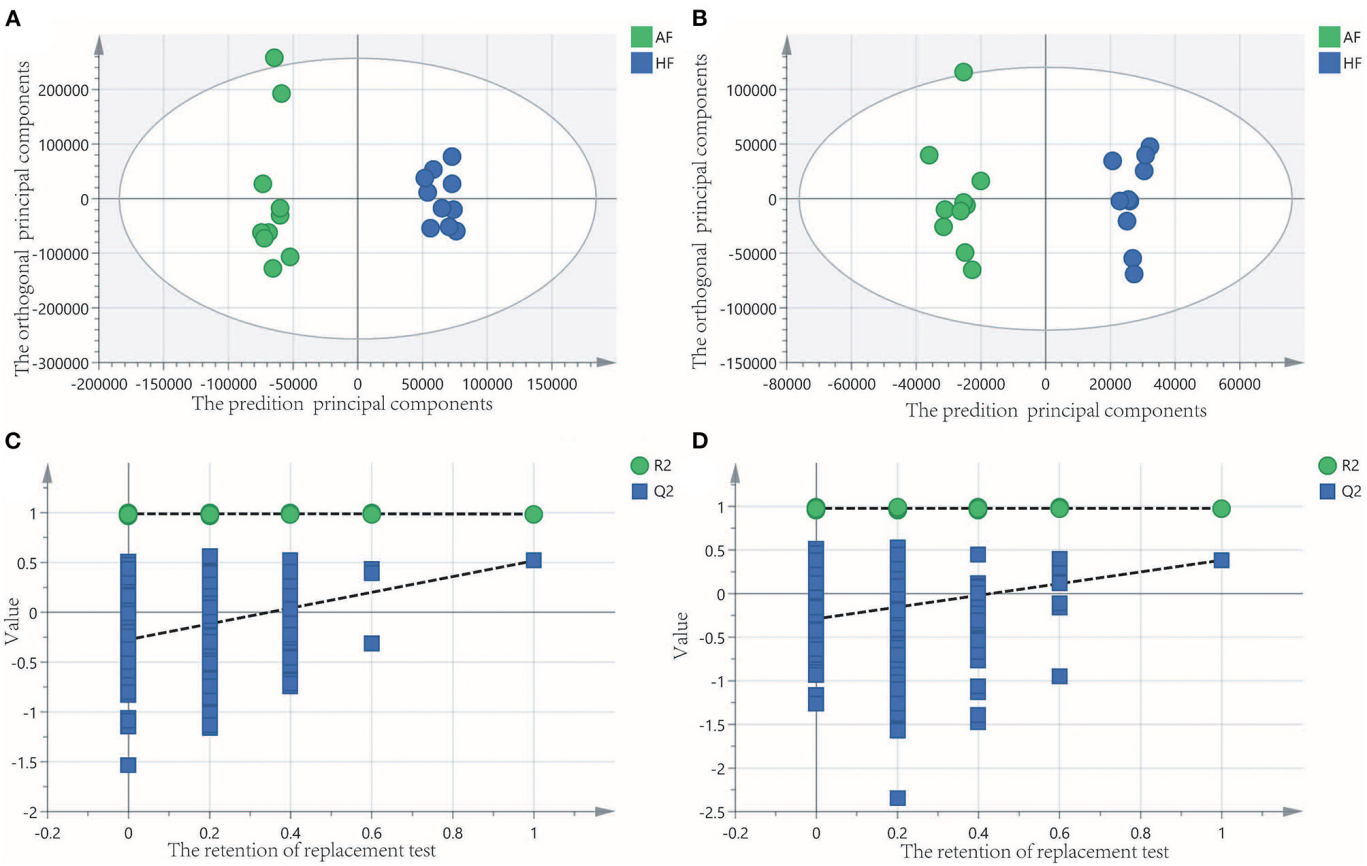
Orthogonal partial least squares discriminant analysis (OPLS-DA) of score and permutation test plot for the healthy follicle (HF) and atretic follicle (AF) groups. **(A,C)** were in the positive ion mode; **(B,D)** were in the negative ion mode. The intercept limit of Q2 was calculated by the regression line, which was the plot of Q2 from the permutation test in the OPLS-DA model.

**Figure 5:**
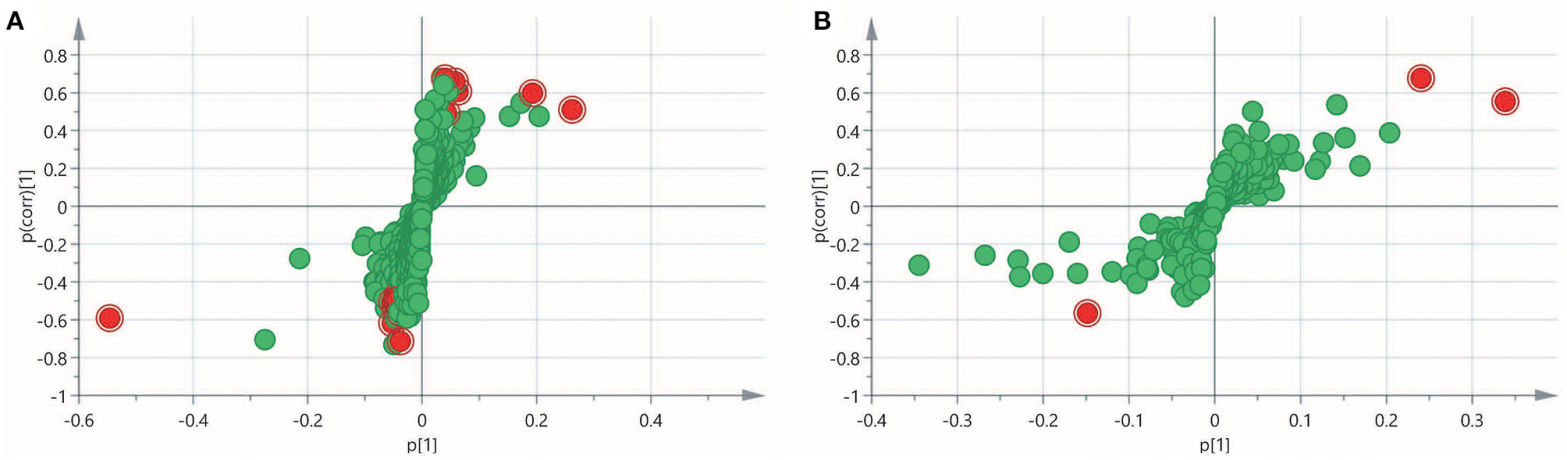
Variable importance in projection (VIP) score plot for the healthy follicle (HF) and atretic follicle (AF) groups. **(A)** The VIP score plot for the two groups analyzed in the positive ion mode. **(B)** The VIP score plot for the two groups analyzed in the negative ion mode. p[1] was the load of each substance under the first principal component; p(corr)[1] was the correlation between each substance and the first principal component. The red plots were the differential metabolites.

**Figure 6:**
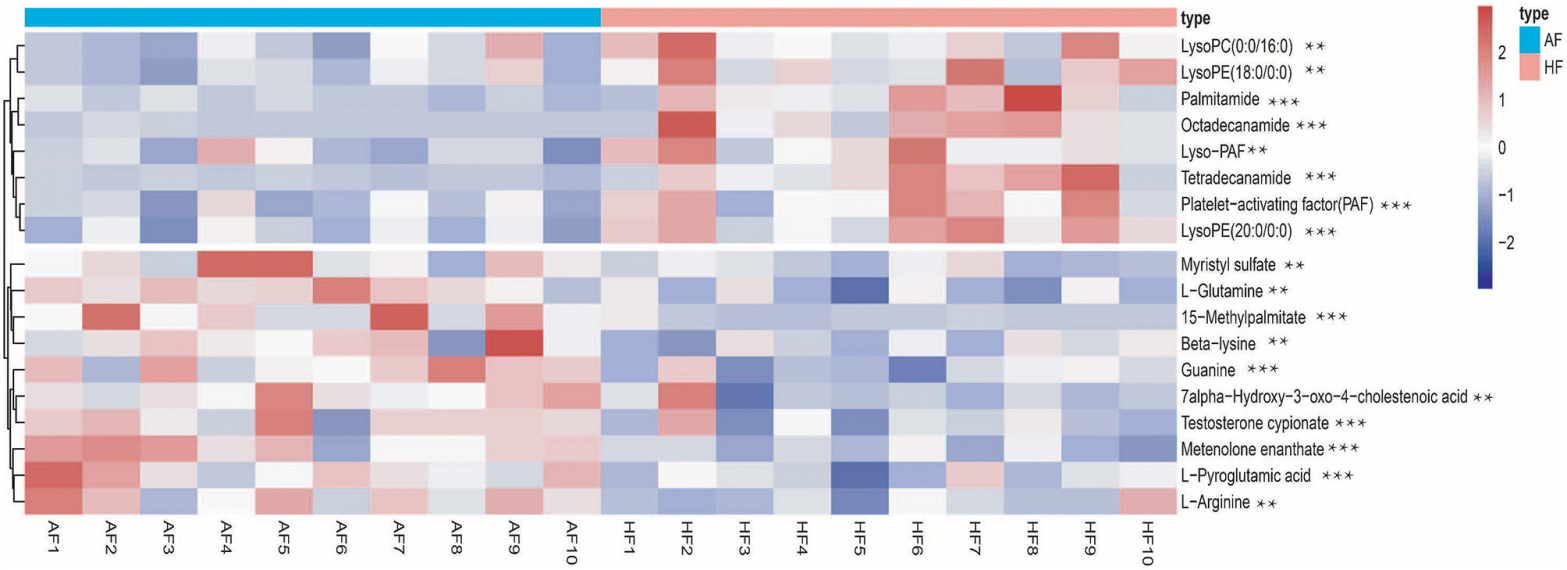
Heatmap of the different metabolites in the follicular fluid of the healthy follicle (HF) and atretic follicle (AF) groups. ** significant difference (*p* < 0.05); *** highly significant difference (*p* < 0.01).

To obtain an overview of the FF metabolic characteristics of Bama Xiang pig follicular atresia, non-targeted metabolic profiling was conducted using the UPLC–MS/MS method. In this experiment, 3,044 peaks in the positive and negative ion modes were obtained. After filtering using the mzCloud database, HMDB database, total ion chromatograms, molecular weight, retention time, peak area, and peak intensity, a total of 994 metabolites were matched. The PCA showed that the R2X model interpretation rates for the two groups under the positive and negative ion mode conditions were 0.62 and 0.679, respectively. Four quality control (QC) samples gathered together not only in the positive PCA score plot but also in the negative PCA score plot (Figure 3). The OPLS-DA showed that samples from the two groups were well-separated, and each tended to cluster in the same group (Figures 4A,B). In the positive ion mode of OPLS-DA, R2Y and Q2 were 0.986 and 0.517, respectively, whereas in the negative ion mode, R2Y and Q2 were 0.979 and 0.38, respectively. The intercepts of permutation tests in the positive ion mode and negative ion mode were −0.257 and −0.353, respectively (Figures 4C,D). A total of 18 metabolites, including l-glutamine, beta-lysine, testosterone cypionate, and l-arginine, were identified as DMs between the HF and AF groups (Table 4, Figure 5). The levels of eight DMs, including palmitamide, octadecanamide, and tetradecanamide, were higher in the HF than in the AF (*p* < 0.01), and those of 10 DMs, including l-glutamine, l-arginine, and guanine, were higher in the AF than in the HF (*p* < 0.01) (Figure 6). Using MetaboAnalyst 4.0 to analyze the 18 DMs showed that they were completely enriched in 12 metabolic pathways, such as arginine biosynthesis, alanine, aspartate, and glutamate metabolism, and primary bile acid (BA) biosynthesis (Table 5).

**Table 4:** Information of 18 differential metabolites.

**Name**	**MW (Da)**	**RT (min)**	**FC**	**VIP**	**Ion mode**
15-Methylpalmitate	287.28188	14.06	0.21	14.7694	P
LysoPC (0:0/16:0)	495.33163	17.93	1.59	7.09451	P
Platelet-activating factor (PAF)	523.36237	18.781	2.22	5.17624	P
Palmitamide	255.25574	18.496	4.69	1.68894	P
Octadecanamide	283.28693	19.172	9.71	1.56161	P
L-Glutamine	146.06888	0.744	0.67	1.4515	P
7alpha-Hydroxy-3-oxo-4-cholestenoic acid	430.30736	16.088	0.67	1.40831	P
L-Pyroglutamic acid	129.04248	0.58	0.78	1.25267	P
Beta-lysine	146.10531	0.538	0.72	1.21497	P
Testosterone cypionate	412.2969	17.669	0.65	1.20554	P
Guanine	151.04914	1.451	0.77	1.16932	P
Lyso-PAF	481.35226	18.524	1.53	1.14234	P
Tetradecanamide	227.22444	17.649	6.99	1.05941	P
L-Arginine	174.11136	1.288	0.70	1.04384	P
Metenolone enanthate	414.31281	17.8	0.47	1.00324	N
LysoPE(18:0/0:0)	481.31663	17.914	1.72	5.40505	N
LysoPE(20:0/0:0)	509.34799	18.872	2.18	3.83837	N
Myristyl sulfate	294.18663	17.563	0.65	2.37024	N

*Fold changes (FC) were calculated as the peak area average levels in the HF group relative to those in the AF group. A fold changes > 1 indicates relatively higher concentration in the HF group, whereas a fold change of < 1 indicates a concentration lower than that in the AF group. MW, molecular weight; RT, retention time; VIP, variable importance in projection; P, positive ion mode; N, negative ion mode*.

**Table 5:** Results of metabolic pathway enrichment analysis.

**Pathway name**	**Match status**	***P***	**Impact**	**Details**
Arginine biosynthesis	2/14	0.0032727	0.07614	KEGG
Aminoacyl-tRNA biosynthesis	2/48	0.036071	0.0	KEGG
D-Glutamine and D-glutamate metabolism	1/6	0.038151	0.0	KEGG
Nitrogen metabolism	1/6	0.038151	0.0	KEGG
Purine metabolism	2/65	0.062718	0.01281	KEGG
Ether lipid metabolism	1/20	0.12213	0.08434	KEGG
Glutathione metabolism	1/28	0.16709	0.00709	KEGG
Alanine, aspartate and glutamate metabolism	1/28	0.16709	0.11378	KEGG
Glyoxylate and dicarboxylate metabolism	1/32	0.18879	0.0	KEGG
Arginine and proline metabolism	1/38	0.22038	0.05786	KEGG
Pyrimidine metabolism	1/39	0.22554	0.0	KEGG
Primary bile acid biosynthesis	1/46	0.26078	0.0	KEGG

*Metabolic pathway analysis of differential metabolites*.

## Discussion

First, we identified HFs and AFs based on the appearance of the follicles. We then measured the concentration of hormones in FF and gene expression in GCs to prove the identification accuracy of HFs and AFs. Similar to the research on *VEGFA* (28) in follicular atresia, the expression of *VEGFA* was approximately two times higher in the HF than in the AF in our study. Furthermore, HFs appeared to have more blood vessels than AFs. This result indicated that the expression of *VEGFA* gene in HF plays a key role in blood vessel generation to maintain the health of the follicles. Meanwhile, the expression of *BAX* (29), *BCL2* (29, 30), *CASPASE3* (30), and *CYP19A1* (31) and the concentration of E2 (24) and PROG (25) were significantly different, which is consistent with the results of prior research. All these studies illustrated that the grouping of our study was reasonable.

The results of this study suggest that the concentrations of bioactive phospholipid substances, including lysophosphatidylcholine [LysoPC (0-0/16:0)], lysophosphatidylethanolamine [LysoPE (18:0/0-0 and 20:0/0-0)], platelet-activating factor (PAF), and 1-*O*-hexadecyl-lyso-sn-glycero-3-phosphocholine (Lyso-PAF), are lower in the AF than in the HF. *In vivo*, LPC and LPE can be converted into PC and PE by acylation, which is mediated by lysophospholipid acyltransferases (LPLATs). PC and PE are the most abundant phospholipids and important structural and functional components of the biological membrane in all mammals (32, 33). The physiological significance of LPE remains unknown, but LPC has been shown to participate in several physiological activities as an effective lipid mediator. LPC is beneficial for GC growth and oocyte maturation by activating the extracellular signal-regulated kinases and nitric oxide (NO) (34). The exogenous addition of LPC could reduce the inhibitory effect of zearalenone on the oocyte maturation process (35). Similar to our study, some recent studies based on FF metabolomics have found more evidence that LPC and LPE are involved in the regulation of follicle development (36, 37). PAF is considered to be the most potent lipid mediator known to date, and the remodeling pathway mediated by LysoPAF is one of the important pathways of PAF biosynthesis (38). PAF has been reported to promote ovulation, facilitate oocyte maturation *in vitro*, enhance extracellular matrix formation around luteal cells, and promote the growth of vascular networks (39). This result is consistent with our findings, as the expression of *VEGFA* was higher in the HF than in the AF, and more blood vessels were observed in HFs. In contrast, estrogen has been proven to cause accumulation of PAF in the ovary by inhibiting the activity of PAF-acetylhydrolase, and the level of PAF is positively correlated with estrogen (40). More importantly, testosterone cypionate as a testosterone can transform to E2 by aromatase reaction (41), and testosterone cypionate level in the AF was significantly higher than that in the HF. Therefore, we hypothesized that testosterone cypionate in the HF was transformed into E2 to promote the accumulation of PAF, which benefited oocyte maturation and maintenance of HFs. However, further research is required to determine the relationship between these factors and follicular atresia. In our results, phospholipid levels were higher in the HF than in the AF. The level of phospholipids that promote follicle development may be achieved by the activation of extracellular signal-regulated kinases and production of NO; the lack of phospholipids may lead to damage of the zona pellucida and GCs, which further results in the deterioration of FF.

An interesting finding of our study was that the levels of amino acids such as l-glutamine, l-pyroglutamic acid, and l-arginine in FF samples were increased by atresia. However, studies have shown that l-pyroglutamic acid can be converted into glutamate to synthesize glutathione (GSH). Reduced GSH is a powerful antioxidant that protects GCs against oxidative stress caused by reactive oxygen species, maintaining normal mitochondrial function and inhibiting cell apoptosis (42). l-arginine improves oocyte maturation and embryo development by increasing NO production (43). These amino acids appear to have a positive effect on follicle development; however, they are upregulated in the FF of AFs, which seems contradictory. However, the concentration of amino acids was not higher. A higher concentration of amino acids means that the balance of osmotic pressure is affected, and the disruption of normal amino acid metabolism may damage the balance of intracellular osmotic pressure and ammonia level in FF, which may injure the oocyte and GCs (44). Therefore, disorders of amino acid metabolism may be one of the factors contributing to follicular atresia in pigs.

Additionally, this study revealed differences in BA metabolism. We compared the FF from HFs and AFs and found that in the FF from AFs, cholic acid (primary BA, *p* < 0.1) and its synthetic precursor 7-hydroxy-3-oxy-4-cholesteric acid were significantly upregulated, while secondary BAs, including glycoursodeoxycholic acid (*p* < 0.1) and glycodeoxycholic acid (*p* < 0.1), were downregulated. BAs are cytotoxic molecules, and excessive BAs have been proven to disrupt the mitochondrial function of cells, induce oxidative stress, and ultimately damage the ovary (45). The aliphatic side chain of primary BAs is conjugated to an amide linkage (N-acyl amidation) with glycine or taurine to form secondary BAs and reduce BA toxicity (46). Our results indicated that cholic acid synthesis was increased and the degradation was decreased, which led to the accumulation of cholic acid in AFs. Finally, the disruption of cholic acid metabolism may damage the follicles by inducing oxidative stress.

## Conclusion

In summary, this study analyzed the different metabolic profiles of FF between HFs and AFs in Bama Xiang pigs. Our results revealed that the metabolic characteristics of porcine AFs are lower levels of lipids (such as phospholipids) and higher levels of amino acids and BAs than those in HFs. Disorders of amino acid metabolism and cholic acid metabolism may contribute to follicular atresia in pigs.

## Data Availability Statement

The raw data supporting the conclusions of this article will be made available by the authors, without undue reservation.

## Ethics Statement

The animal study was reviewed and approved by the College of Animal Science & Technology, Guangxi University (Ethics approval reference number: GXU-2021-055).

## Author Contributions

JM, LS, and JC designed the study, conducted the experiments, and drafted the paper. YL, YW, and GQ conducted parts of the experiments. JL and GL revised the paper. All authors have read and agreed to the published version of the manuscript.

## Conflict of Interest

The authors declare that the research was conducted in the absence of any commercial or financial relationships that could be construed as a potential conflict of interest.

## Publisher's Note

All claims expressed in this article are solely those of the authors and do not necessarily represent those of their affiliated organizations, or those of the publisher, the editors and the reviewers. Any product that may be evaluated in this article, or claim that may be made by its manufacturer, is not guaranteed or endorsed by the publisher.

## References

[1] Meng L Teerds K Tao J Wei H Jaklofsky M Zhao Z. Characteristics of circular RNA expression profiles of porcine granulosa cells in healthy and atretic antral follicles. Int J Mol Sci. (2020) 21:5217. 10.3390/ijms21155217 32717899 PMC7432752

[2] Shan X Yu T Yan X Wu J Fan Y Guan X. Proteomic analysis of healthy and atretic porcine follicular granulosa cells. J Proteomics. (2021) 232:104027. 10.1016/j.jprot.2020.104027 33130110

[3] Racine C Genêt C Bourgneuf C Dupont C Plisson-Petit F Sarry J. New anti-müllerian hormone target genes involved in granulosa cell survival in women with polycystic ovary syndrome. J Clin Endocrinol Metab. (2021) 106:e1271–89. 10.1210/clinem/dgaa879 33247926

[4] Muthulakshmi S Hamideh PF Habibi HR Maharajan K Kadirvelu K Mudili V. Mycotoxin zearalenone induced gonadal impairment and altered gene expression in the hypothalamic-pituitary-gonadal axis of adult female zebrafish (Danio rerio). J Appl Toxicol. (2018) 38:1388–97. 10.1002/jat.3652 29923290

[5] He Y Deng H Jiang Z Li Q Shi M Chen H. Effects of melatonin on follicular atresia and granulosa cell apoptosis in the porcine. Mol Reprod Dev. (2016) 83:692–700. 10.1002/mrd.22676 27391761

[6] Socha JK Hrabia A. Response of the chicken ovary to GH treatment during a pause in laying induced by fasting. Domest Anim Endocrinol. (2019) 69:84–95. 10.1016/j.domaniend.2019.05.001 31382237

[7] Chu YL Xu YR Yang WX Sun Y. The role of FSH and TGF-beta superfamily in follicle atresia. Aging. (2018) 10:305–21. 10.18632/aging.101391 29500332 PMC5892684

[8] Mazerbourg S Monget P. Insulin-like growth factor binding proteins and igfbp proteases: a dynamic system regulating the ovarian folliculogenesis. Front Endocrinol. (2018) 9:134. 10.3389/fendo.2018.00134 29643837 PMC5890141

[9] Bromfield JJ Sheldon IM. Lipopolysaccharide reduces the primordial follicle pool in the bovine ovarian cortex ex vivo and in the murine ovary in vivo. Biol Reprod. (2013) 88:98. 10.1095/biolreprod.112.106914 23515670

[10] Max MC Bizarro-Silva C Bufalo I Gonzalez SM Lindquist AG Gomes RG. *In vitro* culture supplementation of EGF for improving the survival of equine preantral follicles. In Vitro Cell Dev Biol Anim. (2018) 54:687–91. 10.1007/s11626-018-0296-9 30284096

[11] Nishimoto H Hamano S Hill GA Miyamoto A Tetsuka M. Classification of bovine follicles based on the concentrations of steroids, glucose and lactate in follicular fluid and the status of accompanying follicles. J Reprod Dev. (2009) 55:219–24. 10.1262/jrd.20114 19194065

[12] Patti GJ Yanes O Siuzdak G. Metabolomics: the apogee of the omic triology. Nat rev Mol Cell Bio. (2013) 13:263. 10.1038/nrm3314 PMC3682684 22436749

[13] Bracewell-Milnes T Saso S Abdalla H Nikolau D Norman-Taylor J Johnson M. Metabolomics as a tool to identify biomarkers to predict and improve outcomes in reproductive medicine: a systematic review. Hum Reprod Update. (2017) 23:723–36. 10.1093/humupd/dmx023 29069503

[14] Sun Z Chang HM Wang A Song J Zhang X Guo J. Identification of potential metabolic biomarkers of polycystic ovary syndrome in follicular fluid by SWATH mass spectrometry. Reprod Biol Endocrinol. (2019) 17:45. 10.1186/s12958-019-0490-y 31186025 PMC6560878

[15] Castiglione MM Iuliano A Schettini S Petruzzi D Ferri A Colucci P. NMR metabolic profiling of follicular fluid for investigating the different causes of female infertility: a pilot study. Metabolomics. (2019) 15:19. 10.1007/s11306-019-1481-x 30830455

[16] Yang Y Adeola AC Xie HB Zhang YP. Genomic and transcriptomic analyses reveal selection of genes for puberty in Bama Xiang pigs. Zool Res. (2018) 39:424–30. 10.24272/j.issn.2095-8137.2018.068 29955027 PMC6085766

[17] Wang LY Lin AG Wang LX Li K Yang GS He RG. Animal Genetic Resources in China Pigs. Beijing: China Agriculture Press (2011). p. 237.

[18] Zhai X Han W Wang M Guan S Qu X. Exogenous supplemental NAD+ protect myocardium against myocardial ischemic/reperfusion injury in swine model. Am J Transl Res. (2019) 11:6066–74. 31632574 PMC6789262

[19] Ning X Yang K Shi W Xu C. Comparison of hypertrophic scarring on a red Duroc pig and a Guangxi Mini Bama pig. Scars Burns Healing. (2020) 6:2059513120930903. 10.1177/2059513120930903 32637158 PMC7318807

[20] Guo M Liu J Guo F Shi J Wang C Bible PW. Panax quinquefolium saponins attenuate myocardial dysfunction induced by chronic ischemia. Cell Physiol Biochem. (2018) 49:1277–88. 10.1159/000493407 30205393

[21] Zhu TY Ai J Nie CH Zhou GH Chen XH Zhang YL. Feasibility of computed tomography-guided irreversible electroporation for porcine kidney ablation. J Cancer Res Ther. (2020) 16:1125–8. 10.4103/jcrt.JCRT_594_19 33004758

[22] Zhang J Liu Y Yao W Li Q Liu H Pan Z. Initiation of follicular atresia: gene networks during early atresia in pig ovaries. Reproduction. (2018) 156:23–33. 10.1530/REP-18-0058 29743261

[23] Kita Y Shindou H Shimizu T. Cytosolic phospholipase A2 and lysophospholipid acyltransferases. Biochim Biophys Acta Mol Cell Biol Lipids. (2019) 1864:838–45. 10.1016/j.bbalip.2018.08.006 30905348

[24] Li M Zhou S Wu Y Li Y Yan W Guo Q. Prenatal exposure to propylparaben at human-relevant doses accelerates ovarian aging in adult mice. Environ Pollut. (2021) 285:117254. 10.1016/j.envpol.2021.117254 33957517

[25] Pargianas M Kosmas I Papageorgiou K Kitsou C Papoudou-Bai A Batistatou A. Follicle inhibition at the primordial stage without increasing apoptosis, with a combination of everolimus, verapamil. Mol Biol Rep. (2020) 47:8711–26. 10.1007/s11033-020-05917-2 33079326

[26] Wang M Bhullar NK. Selection of suitable reference genes for QRT-PCR gene expression studies in rice. Methods Mol Biol. (2021) 2238:293–312. 10.1007/978-1-0716-1068-8_20 33471340

[27] Luo ZZ Shen LH Jiang J Huang YX Bai LP Yu SM. Plasma metabolite changes in dairy cows during parturition identified using untargeted metabolomics. J Dairy Sci. (2019) 102:4639–50. 10.3168/jds.2018-15601 30827559

[28] Gao X Zhang J Pan Z Li Q Liu H. The distribution and expression of vascular endothelial growth factor A (VEGFA) during follicular development and atresia in the pig. Reprod Fertil Dev. (2020) 32:259–66. 10.1071/RD18508 31545934

[29] Zhang T BaSang WD Chang W Huo S Ma X Ju X. Dynamics of apoptosis-related gene expression during follicular development in yak. J Anim Physiol Anim Nutr. (2021). 10.1111/jpn.13527. [Epub ahead of print]. 33899975

[30] Liu H Tian Z Guo Y Liu X Ma Y Du X. Microcystin-leucine arginine exposure contributes to apoptosis and follicular atresia in mice ovaries by endoplasmic reticulum stress-upregulated Ddit3. Sci Total Environ. (2021) 756:144070. 10.1016/j.scitotenv.2020.144070 33288253

[31] Li Q Du X Liu L Liu H Pan Z Li Q. Upregulation of miR-146b promotes porcine ovarian granulosa cell apoptosis by attenuating CYP19A1. Domest Anim Endocrinol. (2021) 74:106509. 10.1016/j.domaniend.2020.106509 32653739

[32] Van der Veen JN Kennelly JP Wan S Vance JE Vance DE Jacobs RL. The critical role of phosphatidylcholine and phosphatidylethanolamine metabolism in health and disease. Biochim Biophys Acta Biomembr. (2017) 1859:1558–72. 10.1016/j.bbamem.2017.04.006 28411170

[33] Kim EA Kim JA Park MH Jung SC Suh SH Pang MG. Lysophosphatidylcholine induces endothelial cell injury by nitric oxide production through oxidative stress. J Matern Fetal Neonatal Med. (2009) 22:325–31. 10.1080/14767050802556075 19089771

[34] Sriraman V Modi SR Bodenburg Y Denner LA Urban RJ. Identification of ERK and JNK as signaling mediators on protein kinase C activation in cultured granulosa cells. Mol Cell Endocrinol. (2008) 294:52–60. 10.1016/j.mce.2008.07.011 18694803

[35] Lai FN Liu XL Li N Zhang RQ Zhao Y Feng YZ. Phosphatidylcholine could protect the defect of zearalenone exposure on follicular development and oocyte maturation. Aging. (2018) 10:3486–506. 10.18632/aging.101660 30472698 PMC6286824

[36] Wen X Kuang Y Zhou L Yu B Chen Q Fu Y. Lipidomic components alterations of human follicular fluid reveal the relevance of improving clinical outcomes in women using progestin-primed ovarian stimulation compared to short-term protocol. Med Sci Monit. (2018) 24:3357–65. 10.12659/MSM.906602 29783268 PMC5989624

[37] Song J Wang X Guo Y Yang Y Xu K Wang T. Novel high-coverage targeted metabolomics method (SWATHtoMRM) for exploring follicular fluid metabolome alterations in women with recurrent spontaneous abortion undergoing in vitro fertilization. Sci Rep. (2019) 9:10873. 10.1038/s41598-019-47370-7 31350457 PMC6659694

[38] Lordan R Tsoupras A Zabetakis I Demopoulos CA. Forty years since the structural elucidation of platelet-activating factor (PAF): historical, current, and future research perspectives. Molecules. (2019) 24:4414. 10.3390/molecules24234414 31816871 PMC6930554

[39] Vandenberghe L Heindryckx B Smits K Szymanska K Ortiz-Escribano N Ferrer-Buitrago M. Platelet-activating factor acetylhydrolase 1B3 (PAFAH1B3) is required for the formation of the meiotic spindle during in vitro oocyte maturation. Reprod Fertil Dev. (2018) 30:1739–50. 10.1071/RD18019 30008286

[40] Pritchard PH. The degradation of platelet-activating factor by high-density lipoprotein in rat plasma. Effect of ethynyloestradiol administration. Biochem J. (1987) 246:791–4. 10.1042/bj2460791 3689334 PMC1148348

[41] Colleluori G Aguirre L Napoli N Qualls C Villareal DT Armamento-Villareal R. Testosterone therapy effects on bone mass and turnover in hypogonadal men with type 2 diabetes. J Clin Endocrinol Metab. (2021). 10.1210/clinem/dgab181. [Epub ahead of print]. 33735389 PMC8599870

[42] Circu ML Aw TY. Glutathione and apoptosis. Free Radic Res. (2008) 42:689–706. 10.1080/10715760802317663 18671159 PMC3171829

[43] Zeng X Mao X Huang Z Wang F Wu G Qiao S. Arginine enhances embryo implantation in rats through PI3K/PKB/mTOR/NO signaling pathway during early pregnancy. Reproduction. (2013) 145:1–7. 10.1530/REP-12-0254 23081893

[44] Wang W Zhang W Liu J Sun Y Li Y Li H. Metabolomic changes in follicular fluid induced by soy isoflavones administered to rats from weaning until sexual maturity. Toxicol Appl Pharmacol. (2013) 269:280–9. 10.1016/j.taap.2013.02.005 23454585

[45] Ommati MM Farshad O Niknahad H Arabnezhad MR Azarpira N Mohammadi HR. Cholestasis-associated reproductive toxicity in male and female rats: the fundamental role of mitochondrial impairment and oxidative stress. Toxicol Lett. (2019) 316:60–72. 10.1016/j.toxlet.2019.09.009 31520699

[46] Di Ciaula A Garruti G Lunardi BR Molina-Molina E Bonfrate L Wang DQ. Bile acid physiology. Ann Hepatol. (2017) 16:s4–14. 10.5604/01.3001.0010.5493 29080336

